# Palmitic Acid: Physiological Role, Metabolism and Nutritional Implications

**DOI:** 10.3389/fphys.2017.00902

**Published:** 2017-11-08

**Authors:** Gianfranca Carta, Elisabetta Murru, Sebastiano Banni, Claudia Manca

**Affiliations:** Dipartimento Scienze Biomediche, Università degli studi di Cagliari, Cagliari, Italy

**Keywords:** palmitic acid, *de novo* lipogenesis, lung surfactant, protein palmitoylation, palmitoylethanolamide

## Abstract

Palmitic acid (PA) has been for long time negatively depicted for its putative detrimental health effects, shadowing its multiple crucial physiological activities. PA is the most common saturated fatty acid accounting for 20–30% of total fatty acids in the human body and can be provided in the diet or synthesized endogenously via *de novo* lipogenesis (DNL). PA tissue content seems to be controlled around a well-defined concentration, and changes in its intake do not influence significantly its tissue concentration because the exogenous source is counterbalanced by PA endogenous biosynthesis. Particular physiopathological conditions and nutritional factors may strongly induce DNL, resulting in increased tissue content of PA and disrupted homeostatic control of its tissue concentration. The tight homeostatic control of PA tissue concentration is likely related to its fundamental physiological role to guarantee membrane physical properties but also to consent protein palmitoylation, palmitoylethanolamide (PEA) biosynthesis, and in the lung an efficient surfactant activity. In order to maintain membrane phospholipids (PL) balance may be crucial an optimal intake of PA in a certain ratio with unsaturated fatty acids, especially PUFAs of both n-6 and n-3 families. However, in presence of other factors such as positive energy balance, excessive intake of carbohydrates (in particular mono and disaccharides), and a sedentary lifestyle, the mechanisms to maintain a steady state of PA concentration may be disrupted leading to an over accumulation of tissue PA resulting in dyslipidemia, hyperglycemia, increased ectopic fat accumulation and increased inflammatory tone via toll-like receptor 4. It is therefore likely that the controversial data on the association of dietary PA with detrimental health effects, may be related to an excessive imbalance of dietary PA/PUFA ratio which, in certain physiopathological conditions, and in presence of an enhanced DNL, may further accelerate these deleterious effects.

## Introduction

Palmitic acid (16:0, PA) is the most common saturated fatty acid found in the human body and can be provided in the diet or synthesized endogenously from other fatty acids, carbohydrates and amino acids. PA represents 20–30% of total fatty acids (FA) in membrane phospholipids (PL), and adipose triacylglycerols (TAG) (Carta et al., 2015). On average, a 70-kg man is made up of 3.5 Kg of PA. As the name suggests, PA is a major component of palm oil (44% of total fats), but significant amounts of PA can also be found in meat and dairy products (50–60% of total fats), as well as cocoa butter (26%) and olive oil (8–20%). Furthermore, PA is present in breast milk with 20–30% of total fats (Innis, 2016). The average intake of PA is around 20–30 g/d representing about 8–10 en% (Sette et al., 2011). PA tissue content seems to be controlled around a well-defined concentration, since changes in its intake do not influence significantly its tissue concentration (Innis and Dyer, 1997; Song et al., 2017), because the intake is counterbalanced by PA endogenous biosynthesis via *de novo* lipogenesis (DNL). Particular physiopathological conditions and nutritional factors may strongly induce DNL, resulting in increased tissue content of PA and disrupted homeostatic control of its tissue concentration (Wilke et al., 2009). However, under normal physiological conditions, PA accumulation is prevented by enhanced delta 9 desaturation to palmitoleic acid (16:1n−7, POA) and/or elongation to stearic acid (SA) and further delta 9 desaturation to oleic acid (18:1, OA) (Strable and Ntambi, 2010; Silbernagel et al., 2012). The tight homeostatic control of PA tissue concentration is likely related to its fundamental physiological role in several biological functions. Particularly in infants PA seems to play a crucial role as recently thoroughly revised by Innis (Innis, 2016). The disruption of PA homeostatic balance, implicated in different physiopathological conditions such as atherosclerosis, neurodegenerative diseases and cancer, is often related to an uncontrolled PA endogenous biosynthesis, irrespective of its dietary contribution.

## Endogenous PA biosynthesis by DNL and metabolic outcomes

FA synthesis starts with citrate conversion to acetyl-CoA and then malonyl-CoA, which is then elongated to form palmitate and other FA. Key enzymes in this process are acetyl-CoA carboxylase (ACC), which catalyzes the DNL limiting step reaction, and the FA synthase (FAS). The main sources of citrate for DNL are glucose and glutamine-derived α-ketoglutarate (α-KG), especially under hypoxia or disruption of the mitochondrial oxidative machinery (Vernieri et al., 2016). Carbohydrate feeding, beyond the body capacity to store it as glycogen or use it as energy substrate, promotes DNL by inducing a raise of insulin and substrate availability (Cohen et al., 2011). Insulin stimulates the transcription factor Sterol Regulatory Element-Binding Proteins-1c (SREBP-1c) which up-regulates the enzymes that catalyze lipogenesis (Horton et al., 2002). Glucose also stimulates lipogenesis by activating the transcription factor of carbohydrate-binding protein (ChREBP) (Uyeda and Repa, 2006). Like SREBP-1c, ChREBP induces different genes involved in fatty acid biosynthesis (Uyeda and Repa, 2006). Unlike glucose, fructose, being taken up almost totally by the liver (Tappy and Le, 2010), cannot be used for glycogen biosynthesis and is promptly converted to glyceraldehyde-3-phosphate, providing a substrate for DNL. The yearly consumption of fructose has gradually increased and likely contributes to the raise of non-alcoholic fatty liver disease (NAFLD) (Cohen et al., 2011).

DNL is a highly conserved pathway also present in invertebrate species where the survival capability seems to be related to their ability to store energy reserves as fat from different sugars in the diet (Biolchini et al., 2017).

In humans, in post-prandial state, dietary carbohydrates could provide a potential source of liver FA through the DNL process (Donnelly et al., 2005). Most of the studies conducted on fasting subjects showed however that the contribution of DNL to the total pool of hepatic FA was modest in healthy subjects in a regular diet. Whereas, conditions such as obesity, insulin resistance as well as NAFLD, DNL has been found markedly induced, heavily contributing to liver fat deposition and changes in fatty acid composition (Marques-Lopes et al., 2001). Moreover, differences in DNL rates found in different studies were related to fasted or post-prandial state, since DNL is suppressed by fasting (Schwarz et al., 1995). Another confounding factor is the role of fatty acids binding proteins (FABP), indeed Cao et al. (2008), found that FABP greatly determined the impact of dietary fat on adipose lipid composition and metabolism. In fact, in the absence of these fatty acid chaperones, adipose tissue markedly relies on DNL.

From fasting studies in patients with NAFLD, excessive uptake of FA in the liver could be attributed to insulin resistance in adipose tissue (Fabbrini et al., 2008). Lambert and colleagues observed that in patients with non-alcoholic fatty liver (NAFL) DNL rates were correlated to the amount of liver fat deposition. Triacylglycerol PA, from DNL, was also increased, and the values were independently associated to intrahepatic TAG (Lambert et al., 2014).

When DNL is increased by short-term supply of high carbohydrate foods in humans, a central enzyme, stearoyl-CoA desaturase (SCD), is regulated in parallel with the DNL pathway (Chong et al., 2008; Collins et al., 2010). In human adipocytes, PA derived from DNL is preferably elongated and desaturated with respect to the exogenous PA, suggesting that DNL can serve as a key regulator in concert with elongation and desaturation to maintain cell membrane fluidity and insulin sensitivity (Collins et al., 2010), lowering PA tissue concentration.

An increase of SCD1 activity in obese subjects has been associated to lower fat oxidation and higher fat storage (Hulver et al., 2005). However, it has also been reported a link between SCD1 content and insulin sensitivity in humans (Peter et al., 2009), and a protection against fat-induced insulin resistance in rat muscle cells was observed following a transient increase of SCD1 content (Pinnamaneni et al., 2006; Bergman et al., 2010). This may suggest that desaturation of *de novo* synthesized FA may be required to modulate TAG biosynthesis and prevent lipotoxic effects by excessive saturated fat accumulation (Collins et al., 2010), and consequent cellular dysfunction involved in the metabolic syndrome (Brookheart et al., 2009; Cnop et al., 2012). It is still debated whether POA, the SCD1 product of PA, is one of the major responsible of such activities, as recently thoroughly reviewed (Souza et al., 2017).

Therefore, overproduction of PA by DNL, activated by physiopathological conditions and chronic nutritional imbalance, leads to a systemic inflammatory response and a metabolic dysregulation, resulting in dyslipidemia, insulin resistance and a dysregulated fat deposition and distribution (Donnelly et al., 2005). Remarkably, a recent study has shown that taste sensitivity to 6-n-propylthiouracil (PROP), a genetic trait of oral chemosensory perception, can influence DNL, and therefore PA red blood cell levels, in association to changes in circulating endocannabinoid levels (Carta et al., 2017). Noteworthy, it has been shown that treatment with endocannabinoid receptor agonist increased DNL in the mouse liver or in isolated hepatocytes (Osei-Hyiaman et al., 2005). Thus, endocannabinoids may influence DNL rate either directly and/or regulating food intake according to PROP sensitivity (Tomassini Barbarossa et al., 2013).

## DNL and cancer

The association of circulating PA levels with cancer development is quite controversial. Association between PA levels in blood fraction in relation to breast cancer risk has been reported in a meta-analysis (Saadatian-Elahi et al., 2004) and a prospective study (Bassett et al., 2016), whereas another prospective study conducted in northern Italy found no association between saturated fatty acids and breast cancer risk (Pala et al., 2001). Since changes in PA intake do not influence significantly its tissue concentration (Innis and Dyer, 1997; Song et al., 2017), it implies that DNL has a central role in cancerogenesis.

DNL is a distinctive pathway of most cancer cells. In fact, although most normal cells use dietary or fatty tissue-derived FA that circulate in the bloodstream either as FFA bound to albumin or as part of lipoproteins, most cancer cells *de novo* synthetize their FA independently from nutrient availability and hormone stimulation (Menendez and Lupu, 2007).

The enhanced DNL in cancer cells is strictly linked to the so-called Warburg effect (Vander Heiden et al., 2009). Warburg found that unlike most normal tissues, cancer cells preferentially use anaerobic glycolysis even in the presence of adequate oxygen supply which would be sufficient to support mitochondrial oxidative phosphorylation, and therefore their metabolism is frequently referred to as “aerobic glycolysis” or Warburg effect (Warburg, 1956). Apparently, Warburg effect seems not to be suitable to meet energy requirements of proliferating cells by not fully taking advantage of complete catabolism of glucose using mitochondrial oxidative phosphorylation to maximize ATP production. In addition, it was shown that it was not due to an impaired mitochondria activity, with consequent compromised aerobic respiration and a reliance on glycolytic metabolism, as first was hypothesized (Warburg, 1956), but later confuted (Weinhouse, 1976; Fantin et al., 2006; Moreno-Sanchez et al., 2007), hypothesizing a different explanation for “aerobic glycolysis” in cancer cells.

Anaerobic glycolysis generates only 2 ATPs per molecule of glucose, while oxidative phosphorylation produces up to 36 ATPs upon total oxidation of one glucose molecule. Therefore, why a metabolism that produces less ATP would be selected for in proliferating cells?

Lower ATP production might be a problem when energy substrates are limited, but not in the case of proliferating mammalian cells, continuously supplied with nutrients from blood, showing high ratios of ATP/ADP and NADH/NAD+ (Christofk et al., 2008; DeBerardinis et al., 2008). Moreover, even small decreases in the ATP/ADP ratio can impair growth, leading cells deficient in ATP to apoptosis (Vander Heiden et al., 1999; Izyumov et al., 2004).

A clarification for the peculiar energy metabolism of proliferating cells is the metabolic requirements that extend beyond ATP.

In fact, proliferating cell must replicate all its cellular contents, which requires nucleotides, amino acids, and lipids biosyntheses. Focusing on DNL, synthesis of PA needs 7 molecules of ATP, 16 carbons from 8 molecules of acetyl-CoA, and 28 electrons from 14 molecules of NADPH.

The production of a 16-carbon fatty acyl chain requires one glucose molecule that, if completely oxidized, can provide five times the ATP necessary, while to produce the required NADPH, 7 glucose molecules are needed. This high asymmetry is only moderately balanced by the consumption of 3 glucose molecules in acetyl-CoA synthesis to satisfy the carbon necessity of the acyl chain. For the cellular proliferation, the amount of the glucose cannot be committed to carbon catabolism for ATP production; indeed, this would give an increase in the ATP/ADP ratio that would severely impair the flux through glycolytic intermediates, reducing the synthesis of the acetyl-CoA and NADPH needed for macromolecular production (Vander Heiden et al., 2009).

If this were the case, the complete conversion of glucose to CO_2_ via oxidative phosphorylation in the mitochondria to maximize ATP production would contrast the requirements of a proliferating cell.

Changes in the correct balance of fuels and/or signal transduction pathways that concern nutrient utilization may trigger the cancer predisposition related to metabolic diseases such as diabetes and obesity (Calle and Kaaks, 2004; Pollak, 2008).

AMP-activated protein kinase (AMPK) is a dominant regulator of metabolism, and key energy sensor of intracellular AMP/ATP ratios. When there is an increase of this ratio (low energy conditions), two molecules of AMP bind, and consequently AMPK is activated (Ellingson et al., 2007). AMPK simultaneously inhibits anabolic pathways, as lipogenesis, and activates catabolic ones such as FA oxidation and glucose uptake (Winder and Hardie, 1999).

**Figure 1:**
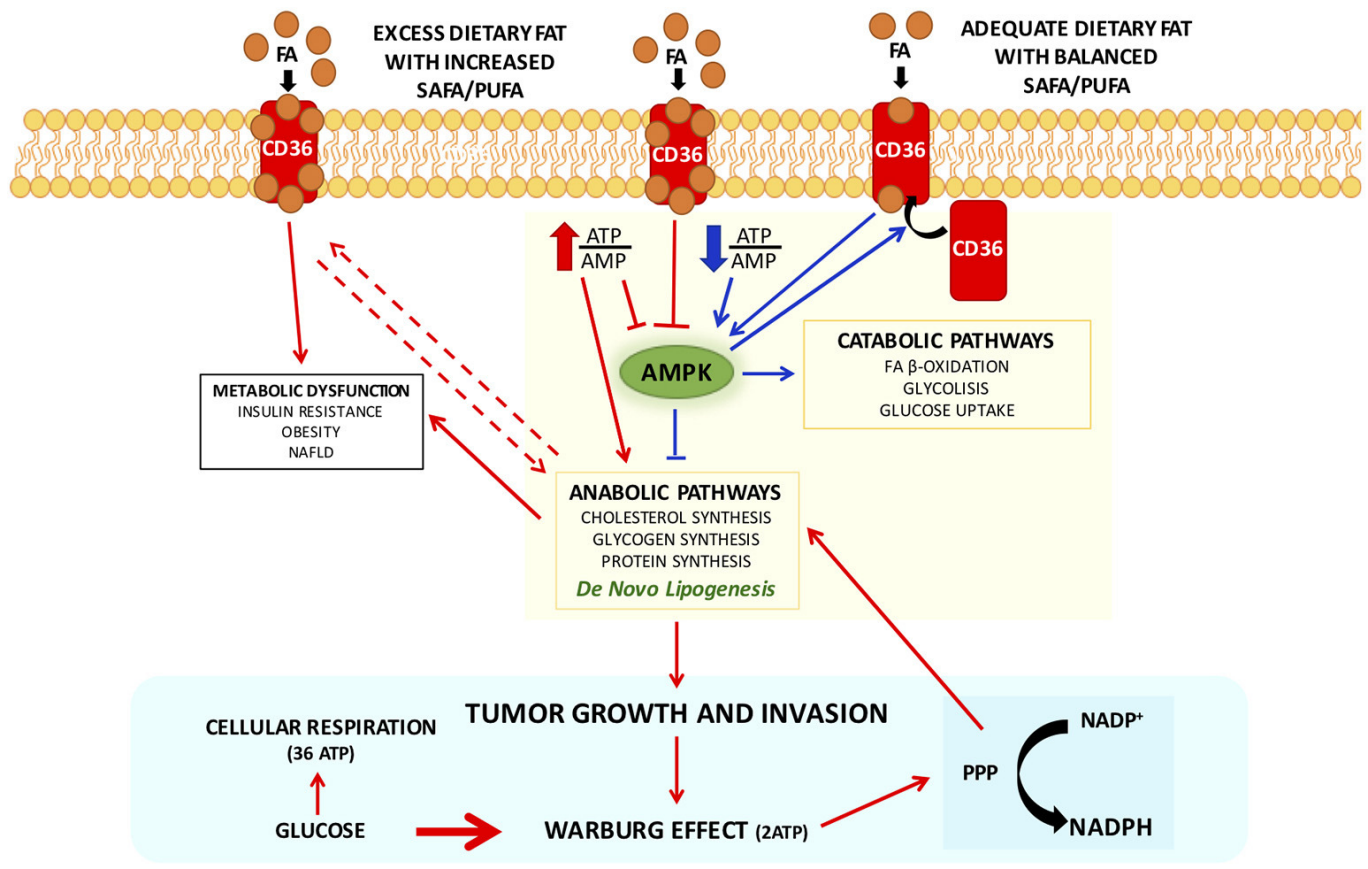
Interplay between CD 36 and AMPK in modulating cellular metabolic pathways with adequate or excess dietary fat, in physiological and pathophysiological conditions. CD36 contributes, under excessive fat supply, to lipid accumulation and metabolic dysfunction signaling (Pepino et al., 2014) and might influence AMPK activation (Samovski et al., 2015). In presence of excess exogenous FA concentrations CD36 is diysfunctional and suppresses AMPK, and might contribute to the reported association of CD36 variants with metabolic complications of obesity in humans. Physiologically adequate CD36-FA interactions activates AMPK (Samovski et al., 2015) contributing to the maintenance of cellular fatty acids homeostasis. Pointing out on the importance of CD36 in cellular fatty acid homeostasis, CD36-deficient mice are resistant to alcohol- and high-carbohydrate-induced hepatic steatosis (Clugston et al., 2014). Therefore, CD36, in particular dysmetabolic conditions, by suppressing AMPK activation, may enhance DNL and thereby promote cancer cell proliferation. Indeed, it has been recently shown that cancer cells that express high levels of the fatty acid receptor CD36 and lipid metabolism genes, are unique in their ability to initiate metastasis in the presence of an excess of dietary fat (Pascual et al., 2017). Arrows in red depict metabolic dysfunction pathways, in blue physiological pathways. Abbreviations: FA, fatty acids; PPP, pentose-phosphate pathway.

Interestingly, CD36, a scavenger receptor that modulates the uptake of long chain fatty acid (LCFA) in high affinity tissues and contributes, under excessive fat supply, to lipid accumulation and metabolic dysfunction signaling (Pepino et al., 2014), might influence AMPK activation (Samovski et al., 2015; Figure 1).

In presence of excess exogenous FA concentrations, CD36 is dysfunctional and suppresses AMPK, and might contribute to the reported association of CD36 variants with metabolic complications of obesity in humans (Figure 1). Physiologically adequate CD36-FA interactions activates AMPK (Samovski et al., 2015) contributing to the maintenance of cellular fatty acids homeostasis.

Not surprisingly, it has been shown that CD36-deficient mice are resistant to alcohol- and high-carbohydrate-induced hepatic steatosis (Clugston et al., 2014). Therefore, CD36, in particular dysmetabolic conditions, by suppressing AMPK activation, may enhance DNL and thereby promote cancer cell proliferation (Figure 1). Indeed, it has been recently shown that cancer cells expressing high levels of the receptor CD36 and lipid metabolism genes, are more likely to initiate metastasis in the presence of an excess of dietary fat (Pascual et al., 2017). Clinically, the presence of CD36+ metastasis-initiating cells correlates with a poor prognosis for different carcinomas, and inhibition of CD36 also affects metastasis, at least in human melanoma- and breast cancer-derived tumors. In addition, activation of CD36 by incubating tumor cells with PA or feeding rats with a diet with 60% of fat resulted in an increased metastatic feature (Pascual et al., 2017). Unfortunately, no other FA or other energy substrates were tested nor a diet enriched in carbohydrates, leaving unresolved whether activation of CD36 is the result of an excess of energy substrate availability. Furthermore, it has been clearly demonstrated that CD36 binds aspecifically most LCFA (Baillie et al., 1996). Future research should be devoted exploring whether CD36 might be also activated by LCFA from sustained DNL during cell proliferation to guarantee the availability of FA for membrane biogenesis. Recently, it has been observed an unexpected association between lipid dysregulation and impaired anti-tumor surveillance, in a model of hepatocellular carcinoma promoted by NAFLD, where disruption of mitochondrial function by linoleic acid induced further oxidative damage, and regulated the loss of intrahepatic CD4+ T lymphocytes. *In vivo* blockade of reactive oxygen species inverted NAFLD-induced hepatic CD4+ T lymphocyte reduction and delayed NAFLD-induced hepatocellular carcinoma (Ma et al., 2016). Thus, it seems to emerge that rather than a single FA, is the imbalance among different FA that may play a role in generating a dysmetabolic cellular milieu which may favor carcinogenesis.

## PA metabolism

One of the key issue on the physiological role of PA is the preservation of a definite tissue concentration and repartition in different lipid classes, which requires a fine regulation of its metabolism. In fact, distribution and metabolism of PA in tissues is strictly controlled.

Of the 20/30 g daily intake 20–30% is beta-oxidized, at a lower rate than the most 18-carbon UFA (Burdge, 2006). Interestingly, in the liver and most bodily tissues about 60–70% of PA is incorporated into PL (Carta et al., 2015).

The liver plays a central role in regulating body concentration of PA, through its desaturation to POA or elongation to SA and further desaturated to OA. As a matter of fact, its plasma concentrations, in physiological conditions, are not significantly influenced by PA dietary intake (Innis and Dyer, 1997; Song et al., 2017), suggesting that the homeostatic control of PA concentration in the liver is regulated by its production via DNL on one side and its desaturation on the other.

Apparently, the priority is to maintain the balance of SAFA/UFA in membrane PL (Abbott et al., 2012). In the liver, the excess of FA is packaged as TAG into VLDL and exported to plasma. Therefore, one might speculate that there is a concerted physiological strategy to maintain the homeostatic balance of PA necessary for physiological functions and in particular to preserve membrane PL physical-chemical properties (Abbott et al., 2012).

The imbalance between SAFA/UFA induces several transcription factors that promote DNL, cholesterol biosynthesis and stearoyl-CoA desaturation (Lee et al., 2015). Therefore, the net result is an increase of hepatic cholesterol biosynthesis with consequent reduction of LDL receptor expression to avoid an excess of cholesterol accumulation in the liver, increase of TAG biosynthesis with a balanced SAFA/UFA ratio and an increase of VLDL and LDL in plasma. As a consequence, one may hypothesize that a prompt physiological response to SAFA/UFA imbalance, may result in a transient hypertriglyceridemia and hypercholesterolemia and a moderate increase of TAG deposition in the liver. However, concurrently physiopathological conditions, such as insulin resistance and chronic nutritional imbalance, may persistently challenge the homeostatic control of PA resulting in an excess of TAG deposition in liver and chronic hypercholesterolemia, hypertriglyceridemia and hyperglycemia, impairing body fat deposition and distribution, which through dysregulated production of adipokines and proinflammatory cytokines trigger a series of events that may be prodromal for chronic pathological conditions such as obesity, cardiovascular disease, type 2 diabetes and cancer. As a matter of fact, several studies have provided consistent evidence that replacing SAFA with PUFA, but not carbohydrates, which may further lead to an increased tissue PA levels via DNL, is beneficial for coronary heart disease (Siri-Tarino et al., 2010).

## Membrane composition

The capacity to store energy in the form of TAG may be envisaged as a later evolutionary change, realized by eukaryotes, of the processes developed from prokaryotes ability to synthetize membrane lipids, as proved by TAG synthesis from modification of PL (Lombard et al., 2012).

Lipid metabolism is most relevant for its involvement in membrane function rather than storage of metabolic energy (Hulbert et al., 2014), providing membranes with essential characteristics for cell division, biological reproduction and intracellular membrane trafficking. Their physical nature also influences the membrane proteins activity allowing some of them to aggregate and others to disperse. Finally, lipids can be chemically modified and they can act as messengers in signal transduction and molecular recognition processes (van Meer et al., 2008).

The membrane PL composition is mainly controlled by feedback regulation of *de novo* PL synthesis. In this regard, membrane-bound transcription factor SREBP seems to control the composition of cellular membrane and consequently regulate lipid synthesis (Dobrosotskaya et al., 2002). *In vitro* studies on rat hepatic cells have shown that *de novo* synthesis yields primarily four molecular species of both phosphatidylcholine (PC) and phosphatidylethanolamine (PE), that are 16:0/18:2, 16:0/18:1, 16:0/22:6, and 18:1/18:2 (Schmid et al., 1995). The other molecular species of PC and PE are formed by their deacylation/reacylation (Schmid et al., 1995). FA desaturation pathway and deacylation/reacylation rapid cycles are mechanisms accountable for the specific FA composition of cell membranes (Pamplona, 2008). Indeed, FA are repetitively removed and substituted at the sn-1 and sn-2 carbons of the glycerol backbone of the lipid, known as “membrane remodeling” (Hulbert et al., 2014). This is a very old process in evolutionary terms and explains why membranes can respond very quickly to environmental change. This is implicated in the temperature-induced modifications in the composition of membrane FA, defined as “homeoviscous adaptation” (Sinensky, 1974).

Remodeling of membrane lipids is also central in the quick removal of oxidized PUFAs. In an experiment where isolated rat hepatocytes were subjected to oxidative stress, Giron-Calle et al. (Giron-Calle et al., 1997) demonstrated that TAG could serve as an expendable FA reserve, providing a limited but very dynamic pool of PUFA for the resynthesis of PL (Giron-Calle et al., 1997).

Numerous studies have shown how dietary FA can influence membrane FA conformation; most of them were merely focused on control and experimental diets. Furthermore, several studies are mainly concerned with a specific FA (often an unusual component of the normal diet of the examined animal) and the studied diet is either lacking, or overloaded with the particular FA. Relatively few studies attempted to examine the normal situation (Hulbert et al., 2014). To quantify the relation between diet and membrane FA composition in rats, Hulbert et al. used 12 isocaloric diets with adequate fat content (25% energy) that were identical, except for their FA profile (which varied widely) (Hulbert et al., 2005; Abbott et al., 2012). FA composition was investigated for brain, heart, liver, skeletal muscle, erythrocytes and plasma PL, as well as adipose tissue and plasma TAG. In different tissues membrane, the relative composition of SAFA remained rather constant over very large variations in the diets. These data suggest that SAFA membrane concentrations are homeostatically regulated irrespective of their dietary intake (Abbott et al., 2012).

Such homeostatic control is also extended to membrane FA composition of hepatic, cardiac, cerebral synaptosome and cerebral myelin membranes of the rat (Vajreswari and Narayanareddy, 1992; Clamp et al., 1997). In contrast, n-6 PUFA content in membranes was more correlated to dietary n-6 PUFA but the highest response was for n-3 PUFA from dietary FA. This suggests that FA composition in membrane will be more influenced by the relative abundance of dietary n-6 and n-3 PUFA than SAFA and MUFA, probably because they cannot be synthesized *de novo* by higher animals (Hulbert et al., 2005).

The FA chain length in membrane lipids influences the physical properties, as fluidity change of state of the membrane bilayer they constitute (Hulbert et al., 2014); alterations of membrane fluidity have been involved in several disease states (Ntambi, 1995, 1999). The fluidity change of state is called phase transition, and the temperature at which it occurs is lower if the hydrocarbon chains are short or have double bonds. Indeed, a shorter chain length reduces the tendency of the hydrocarbon tails to interact with one another, and cis-double bonds produce kinks in the hydrocarbon chains that make them more difficult to pack together, so that the membrane remains fluid at lower temperatures (Alberts et al., 2002). For example, membrane bilayers consisting of 16:0/16:0-PC have a transition temperature of 41°C, while for 18:0/18:1-PC is 6°C, and for 16:0/18:1-PC is −2°C, while for 16:1/16:1-PC is −36°C (Silvius, 1982).

The physical properties that PUFA confer to membrane bilayers and a comparison of the various conformations among PUFA and SAFA have been analyzed by the same author (Feller et al., 2002; Feller, 2008).

In addition, the presence of SAFA into membranes greatly facilitate inclusion of free cholesterol (Simons and Toomre, 2000) that confers to the lipid bilayer a less deformable structure in this region and decreasing the permeability of the bilayer to water-soluble molecules (Alberts et al., 2002).

The ratio among different FA affects membrane fluidity and cell–cell interaction (Ntambi, 1995). Atypical modification of this ratio has been shown to be involved in different physiological and disease states such as diabetes, cardiovascular disease, obesity, hypertension, neurological diseases, immune disorders, cancer, and aging (Spiegelman et al., 1993).

## Lung surfactant

Pulmonary surfactant is a substance essential during breathing and is produced in the lungs by the type II epithelial cells to reduce the surface tension at the air/liquid interface of the alveolus. Natural surfactant is a lipid-protein complex comprising ~90% lipids and 10% proteins. The qualitative and quantitative compositions of the lipids in the surfactant vary between species and according to environmental conditions, such as body temperature (Lang et al., 2005). Surfactant also changes according to physiological constraints, such as the breathing rate or hibernation (Suri et al., 2012), or to pathological situations particularly lung injury (Gunther et al., 1999; Schmidt et al., 2001).

Pulmonary surfactant presents different classes of lipids, such as PL, TAG, cholesterol, and FA and surfactant-associated proteins that extensively interact with PL regulating structure and properties of the lipid film (Yu and Possmayer, 1988; Jobe and Ikegami, 2001; Suresh and Soll, 2001; Ainsworth and Milligan, 2002). The PL composition of surfactant is highly conserved among mammals (Hook, 1991; Sullivan et al., 2002). PC is the main PL covering 80% of surfactant lipids with dipalmitoylphosphatidylcholine (DPPC, 16:0/16:0-PC), the principal surface-active component, accounting for 60% (Thannhauser et al., 1946; Kahn et al., 1995; Creuwels et al., 1997; Jobe and Ikegami, 2001; Suresh and Soll, 2001). It is well known that a monolayer of PL, highly rich in DPPC at the interface, is accountable for surface tension decrease (Goerke, 1974; King, 1984; Notter and Finkelstein, 1984; Possmayer et al., 1984; Goerke and Clements, 1986; Van Golde et al., 1988). The amount of DPPC in the lungs is dependent on their development, with an increase seen at 22 weeks' gestation (Jobe and Ikegami, 2001; Suresh and Soll, 2001).

Other then 16:0/16:0-PC, mammalian surfactant presents near 10% of 16:0/14:0-PC, 30% of 16:0/16:1-PC, and lower concentrations of 16:0/18:1-PC and 16:0/18:2-PC (Jobe and Ikegami, 2001; Suresh and Soll, 2001). Another abundant PL in surfactant is phosphatidylglycerol (PG) that accounts for 7–15% of the total PL and could be involved in alveolar stability as suggested from studies that reported it might control the innate immune response (Possmayer et al., 1984; Hallman et al., 1985; Numata et al., 2010). Other minor PL are PE (~5%), phosphatidylinositol (PI) (~3%), phosphatidylserine (PS), lysoPC, and sphingomyelin (SM); their precise functions are yet to be elucidated (Goerke, 1974; Cockshutt and Possmayer, 1992; Whitsett et al., 2011). SM, which levels decrease with lung maturity, has an inverse proportion relative to PC, indeed the ratio PC/SM is used to determine lung maturity (Jobe and Ikegami, 2001; Suresh and Soll, 2001).

Lung surfactant deficiency and inactivation contribute to the pathophysiology of several important pulmonary diseases. In 1959 Avery and Mead (Avery and Mead, 1959) reported that a deficiency of surfactant in the newborn, often triggered by premature delivery, was responsible for the development of neonatal respiratory distress syndrome (NRDS) (Possmayer, 1988). It was subsequently realized that inactivation of the surfactant system was also involved in the adult form of respiratory distress (ARDS) (Jobe and Ikegami, 1987; Robertson and Lachmann, 1988). The properties of DPPC that make it an ideal surfactant, are its high gel-to-liquid crystalline transition temperature and its capability to be firmly packed into a gel upon compression.

The main biophysical role of the residual surfactant components appears to be the efficient generation and preservation of this DPPC monolayer (Goerke and Gonzales, 1981; Hawco et al., 1981; Wustneck et al., 2005). The two saturated acyl chains permit the lipid to obtain a firmly packed monolayer which can cause these low surface tension values without collapsing. For instance, 30–40% of natural surfactant are unsaturated species of PC, that is a weak surfactant, but, when blended with DPPC creates a more fluid structure which can be more easily adsorbed to the monolayer than pure DPPC (Possmayer et al., 1984; Whitsett et al., 2011). Moreover, the unsaturated PC might be essential in the formation of a lipid reservoir, in their adsorption to the interface, in the regulation of surface tension in the respiratory cycle, and their role in intracellular events. PC relevant amounts of UFA, in balance with DPPC, increase its surfactant properties (Yu and Possmayer, 1988). Consequently, changes in this balance, such as drastic changes in dietary FA, may compromise lung function (Baritussio et al., 1978, 1980; Nohara et al., 1986; Palombo et al., 1994). It has been shown that reduced intake of PUFA by enhancing desaturation of PA decreases DPPC levels in the broncho-alveolar lavage (Kyriakides et al., 1976), particularly during essential fatty acid deficiency (EFAD) (Friedman, 1979).

PA incorporation into lipids is not determined by the absolute intake of PA, but by the ratio among different type of FA. This ratio may have a greater relevance in terms of practical human nutrition in the case of marginal EFAD as it is more likely to exist in the human population with EFAD (particularly in infants). Marginal EFAD is also known to exist in patients with cystic fibrosis (CF) that revealed an elevated level of PE and PG, whereas the degree of saturation of PC was reduced (Hopkins et al., 1963; Kyriakides et al., 1976; Hubbard et al., 1977; Sahu and Lynn, 1977; Friedman and Rosenberg, 1979). Thus, insufficient or biophysically impaired surfactant may play a significant role in the sequence of pathophysiological events that results in reduced function in CF patients (Sahu and Lynn, 1977).

## Protein palmitoylation

Many proteins have been identified as targets of palmitoylation: cell adhesion molecules (Stoeck et al., 2010), CD36 (Thorne et al., 2010) many G-protein-coupled receptors (Maeda et al., 2010), channels and transporters (Crane et al., 2009; Jeffries et al., 2010; Mueller et al., 2010; Du et al., 2017), multiple receptors (Yang et al., 2011; Liu et al., 2012; Lu and Roche, 2012; Oddi et al., 2012; Zheng et al., 2012), receptor ligands (Guardiola-Serrano et al., 2010), but also enzymes (Meckler et al., 2010; Motoki et al., 2012), and chaperones (Lakkaraju et al., 2012).

Considering all these target proteins is evident that S-palmitoylation may have a fundamental role in numerous biological processes such as cell signaling, apoptosis, and tumorigenesis (Tsutsumi et al., 2008; Baekkeskov and Kanaani, 2009).

S-palmitoylation is a covalent post-translational modification of proteins which consists in the attachment of PA to specific cysteines via a thioester bond (Fukata and Fukata, 2010). S-palmitoylated proteins vary in number from ~50 in yeast (Roth et al., 2006) to several hundred in mammals (Martin and Cravatt, 2009; Yang et al., 2010). S-palmitoylation, rises the hydrophobicity of cytoplasmic proteins in a manner similar to other lipid alterations such as myristoylation and prenylation, thereby increasing their affinity for cytosolic membrane surfaces (Yang et al., 2010). However, as opposed to other lipid modifications, S-palmitoylation is reversible; indeed, PA on a protein has a shorter turnover time than the half-life of the protein (Fukata and Fukata, 2010). Reversibility makes this modification an interesting mechanism for the regulation of protein activity and stability, protein-protein interactions, and shuttling of proteins between subcellular compartments when there are modifications of signal transduction (Greaves and Chamberlain, 2007; Linder and Deschenes, 2007). It also allows the fine-tuning of protein function, such as protein phosphorylation or ubiquitination (Blaskovic et al., 2013).

S-palmitoylation of proteins includes: soluble proteins, in which allows their controlled association with membranes, trafficking and domain localization (Rocks et al., 2005; Merrick et al., 2011); the cytosolic domains of membrane proteins (Blaskovic et al., 2013); and transmembrane proteins. The palmitoylation of the latter is less understood, because it often takes place on cysteines adjacent to, or even within, the transmembrane domain (TMD) (Blaskovic et al., 2013). It was suggested that, when palmitoylation occurs on transmembrane proteins, could induce tilting of transmembrane segment to the bilayer surface (Joseph and Nagaraj, 1995).

In proteins without hydrophobic segment, palmitoylation could be implicated in interaction with membrane-bound proteins (Joseph and Nagaraj, 1995).

PA is available in the cell as palmitoyl-CoA at nanomolar concentrations (Faergeman and Knudsen, 1997). Unless palmitoyl-CoA is enriched in specific membrane microdomains (Li et al., 2012), its small concentration doesn't permit spontaneous palmitoylation of proteins and S-palmitoylation is thus mediated by palmitoyltransferases (Fukata and Fukata, 2010).

Several studies focused their attention on the role of palmitoylation of membrane proteins in promoting their association with cholesterol- and sphingolipid-rich membrane microdomains, or lipid rafts (Levental et al., 2010). In particular, the side-chains of PL in rafts are usually highly enriched in SAFA typically PA, leading to a more ordered and less fluid lipid structure than the surrounding membrane, allowing for a close packing of lipids (Calder and Yaqoob, 2007). Rafts seem to act as signaling platforms by bringing together various signaling components facilitating their interaction, i.e., many proteins involved in signal transduction and predominantly found in lipid rafts (Simons and Toomre, 2000). Palmitoylation can increase the affinity of proteins for rafts, but for an overall affinity of the proteins for these microdomains are required other structural features such as prenylation or membrane spans, which prefer a disordered environment (Melkonian et al., 1999). However, palmitoylation-dependent raft association has been shown for cannabinoid receptor (Oddi et al., 2012), and close homolog of adhesion molecule L1 (Tian et al., 2012).

It is not well understood how palmitoylation influences raft association of proteins; a possible explanation may be the affinity of PA for peculiar lipids or lipid domains, as suggested by studies on palmitoylated or not palmitoylated different Ras isoforms (Henis et al., 2009). In the case of transmembrane proteins, however, palmitoylation might lead to changed raft association on the conformation of transmembrane domains (Blaskovic et al., 2013). Indeed, it is thought that the presence of cholesterol makes cholesterol-rich domains thicker than other parts of the membrane, consequently modifications of transmembrane domains tilting through palmitoylation may influence the length of the hydrophobic segments leading to protein partitioning through the membrane thickness (Blaskovic et al., 2013).

## Precursor of palmitoylethanolamide (PEA)

PEA is an endogenous amide of PA that was first purified from lipid fractions of soybeans, egg yolk and peanut meal, and then found in a wide variety of cells, tissues, and body fluids of several animal species and human subjects (Petrosino and Di Marzo, 2017). PEA is used as lipid mediator for its neuroprotective, anti-neuroinflammatory and analgesic activities (Re et al., 2007; Skaper et al., 2015; Iannotti et al., 2016).

In animals, PEA is synthetized through the hydrolysis of its PL precursor, N-palmitoyl-phosphatidylethanolamine, by N-acyl-phosphatidylethanolamine-selective phospholipase D (NAPE-PLD). Indeed, it derives directly by PA esterified in sn-1 position of a donor membrane PL like PC (Hansen, 2013; Tsuboi et al., 2013). Usually, tissue levels of N-acylethanolamides (NAE) seem to display the availability of their local precursor FA in the PL membranes, which are dependent from the diet (Di Marzo et al., 2010; Banni et al., 2011; Piscitelli et al., 2011; Piras et al., 2015).

Different studies have evaluated whether PA tissue concentrations, particularly in membrane PL, are able to influence PEA biosynthesis and whether tissue levels could be influenced by variation in intake of dietary FA. In a previous study, we found that incubation of cultured adipocytes with PA was able to induce a 3-fold increase of PEA (Matias et al., 2008). More recently we found that feeding rats with TAG with PA in the sn-2 position was able to increase PEA in adipose tissue with respect to rats fed fat with PA randomly distributed in TAG. The changes in sn position resulted in a selective incorporation of PA into tissue PL and thereby influencing NAE biosynthesis, which also resulted in a decrease of circulating TNF-alpha after LPS challenge, and to an improved feed efficiency (Carta et al., 2015).

Like other NAE, endogenous PEA is synthetized on demand and exerts its function *in loco* during inflammatory and neurodegenerative disorders to counteract inflammation, neuronal injury and pain. Numerous papers have shown that, as for the endocannabinoids Arachidonylethanolamide (AEA) and 2-arachidonoylglycerol (2-AG), even PEA tissue concentrations are changed in various pathologies (Balvers et al., 2012).

The balance between synthesis and breakdown of PEA finely regulates its tissue concentrations. The degradation of PEA to PA and ethanolamine occurs by the action of two different hydrolytic enzymes. The primary degrading enzyme is fatty acid amide hydrolase (FAAH) (Cravatt et al., 1996; Bisogno, 2008) and, another enzyme has been cloned and found to hydrolyze more specifically PEA, N-acylethanolamine-hydrolyzing acid amidase (NAAA) (Ueda et al., 2001). FAAH is known to be located in the endoplasmic reticulum of cells and it is a membrane-bound enzyme, while NAAA is located inside the lysosomes (Ueda et al., 2010). As PEA is a lipophilic molecule, it can easily flip between the inner leaflet and outer leaflet of the PL bilayer of the plasma membrane, but it will only leave the membrane when there is an appropriate soluble binding protein (Bojesen and Hansen, 2005). In the cytosol, FABP (Kaczocha et al., 2012) and heat-shock proteins (Oddi et al., 2009), may transport PEA to its enzymatic degradation or to target molecules, e.g., PPAR-alpha (Lo Verme et al., 2005), and vanilloid receptor (TRPV1) (De Petrocellis et al., 2001a). In the extracellular fluid, PEA probably binds to serum albumin as do other NAE (Zolese et al., 2000; Bojesen and Hansen, 2003). Early studies of PEA uptake into cells suggested the existence of a facilitated membrane transport, due to the presence of intracellular binding proteins (Bisogno et al., 1997; Jacobsson and Fowler, 2001). Interestingly, the biosynthesis and degradation of PEA, as well as other NAE, in plants, where these compounds exert quite different physiological functions, seem to occur via identical routes and often similar enzymes (Blancaflor et al., 2014).

PEA can act via multiple mechanisms. The first mechanism proposes that PEA can decrease mast-cell degranulation via an “Autacoid Local Inflammation Antagonism” (ALIA) pathway (Iannotti et al., 2016). Another mechanism, known as “entourage effect,” assumes that PEA acts by improving the anti-inflammatory and anti-nociceptive properties of AEA by inhibiting the expression of FAAH enzyme, and consequently its degradation (Di Marzo et al., 2001), or through the stimulation of the expression of diacylglycerol lipases (DAGL), the enzyme responsible of the biosynthesis of 2-AG and may indirectly activate the cannabinoid receptor type 1 (CB1) and CB2 (Di Marzo et al., 2001; Petrosino et al., 2016). Similarly, PEA can indirectly activate TRPV1 channels, other targets for the endocannabinoids (Zygmunt et al., 1999, 2013). In addition, PEA, possibly through allosteric effects, can increase AEA- or 2-AG-induced TRPV1 activation and desensitization (De Petrocellis et al., 2001a; Di Marzo et al., 2001; Petrosino et al., 2016). It has also been shown that PEA can activate TRPV1 channels or up-regulate the expression of CB2 receptors via PPAR-alpha receptors (Ben-Shabat et al., 1998; Lambert and Di Marzo, 1999; De Petrocellis et al., 2001b; Di Marzo et al., 2001; Conti et al., 2002; Smart et al., 2002; Ambrosino et al., 2013). As a final point, the “receptor mechanism” is founded on the ability of PEA to directly stimulates either a not characterized cannabinoid CB2 receptor-like target (Calignano et al., 2001; Costa et al., 2008), or the PPAR-alpha, responsible for the regulation of the anti-inflammatory properties of PEA (Lo Verme et al., 2005). Interestingly, a possible precursor of PEA, the 1-palmitoyl-2-oleoyl-sn-glycerol-3-phosphocholine, has been proposed as the endogenous ligand of PPAR-alpha in the liver (Chakravarthy et al., 2009). An orphan receptor G-protein coupling, GPR55, has been as well shown to be stimulated by PEA as well as AEA by some authors (Ryberg et al., 2007), but not others (Kapur et al., 2009).

As a whole, these findings suggest that PEA may exert its physiological activities through many mechanisms of action, and PA either of dietary origin or formed from DNL, may influence its biosynthesis.

## Conclusions

**Figure 2:**
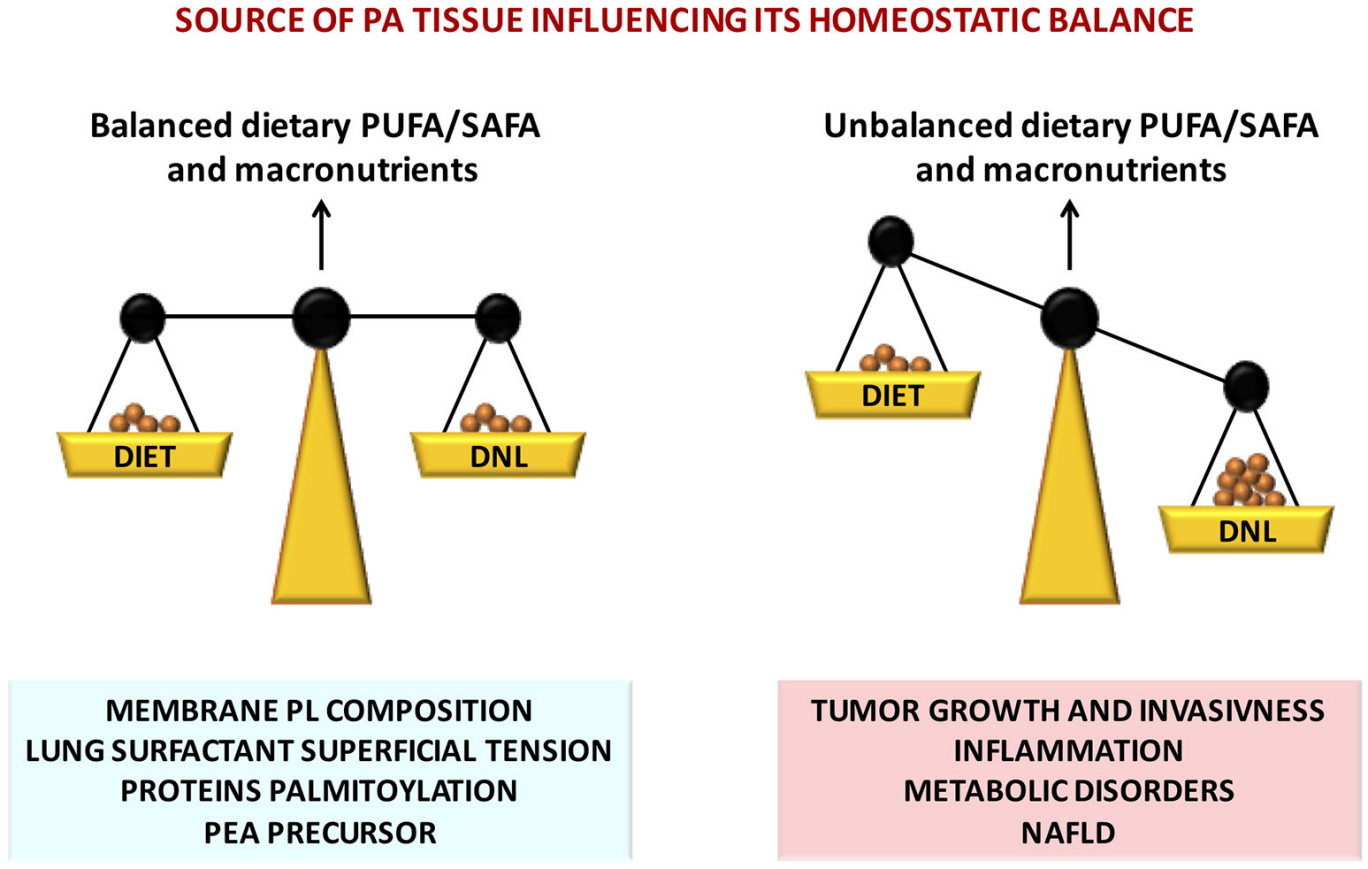
Nutritional factors affecting tissue PA homeostatic balance, disruption of which leads to severe pathophysiological consequences.

PA exerts multiple fundamental biological functions at cellular and tissue levels and its steady concentration is guaranteed by its endogenous biosynthesis by DNL, a metabolic pathway present from the lower steps of the evolutionary scale. However, in the mammals the endogenous biosynthesis may not be sufficient in the long term. Contribution by dietary means is physiologically relevant and may be crucial an optimal intake of PA in a certain ratio with unsaturated fatty acids, especially PUFAs of both n-6 and n-3 families (Figure 2). In fact, FA metabolism appears to be directed to reach an optimal PUFA/SAFA ratio in tissues for maintaining FA membrane PL balance. The optimal FA balance not only guarantees the membrane physical properties but also favors protein palmitoylation, PEA biosynthesis, and in the lung an efficient surfactant activity (Figure 2). The organism is able to cope with short term dietary FA and macronutrient imbalance by modulating lipid metabolism. However, in presence of other factors such as positive energy balance, excessive intake of carbohydrates (in particular mono and disaccharides), and a sedentary lifestyle, the mechanisms to maintain a steady state of PA concentration may be disrupted leading to an over accumulation of tissue PA resulting in dyslipidemia, hyperglycemia, increased ectopic fat accumulation and increased inflammatory tone via toll-like receptor 4 (Figure 2). In these physiopathological conditions, excessive imbalance of PA/PUFA ratio in the diet may further accelerate these deleterious effects. It is therefore likely that the controversial data on dietary PA, and generally on dietary SAFA, may be generated by the different importance given to other variables and, most important, whether it has been taken into account the amount and type of the intake of PUFAs.

## Author contributions

All the Authors contributed to the conception or design of the work, drafting the work and revising it critically for important intellectual content, and approved the version to be published.

### Conflict of interest statement

The authors declare that the research was conducted in the absence of any commercial or financial relationships that could be construed as a potential conflict of interest.
